# Synergistic effects of surfactants and compound acids on the mechanical and microscopic properties of coal

**DOI:** 10.1038/s41598-025-34451-z

**Published:** 2026-01-06

**Authors:** Ruze Liu, Jian Huang, Qingfeng Xu, Ronggang Zhang, Rongyue Li, Fuyao Chu

**Affiliations:** 1 College of Energy and Mining Engineering, Shandong University of Science and Technology, Qingdao, 266590 China; 2 China Energy Shendong Coal Group Co., Ltd., Yulin, 719315 China; 3 Beijing Tanchuang Resource Technology Co., Ltd., Beijing, 100160 China; 4 Shandong Energy Group Luxi Mining Co., Ltd., Heze, 274700 China

**Keywords:** Sodium dodecyl sulfate(SDS), Compound acid, Fractal dimension, Coal dust, Molecular dynamics simulation, Energy science and technology, Engineering, Environmental sciences

## Abstract

This study examines the effects of synergistic treatment with surfactants and compound acids on the mechanical properties (e.g., strength and deformation characteristics) and microscopic features (e.g., pore structure, surface wettability, and chemical composition) of coal. The results indicate that acid treatment weakens coal strength, and the addition of sodium dodecyl sulfate (SDS) further exacerbates this effect. Acidification significantly reduces acoustic emission energy, electromagnetic radiation energy, and infrared radiation temperature released by coal. Moreover, SDS modifies the chemical structure of coal, reducing aromatic content by ~ 30% and fat content by ~ 50% compared to untreated coal. Under the synergistic effect of SDS, acid-induced erosion reaches its maximum. The decrease in fractal dimensions *D*_*W*_ and *D*_*S*_ indicates that SDS promotes coal pore connectivity. Furthermore, molecular dynamics simulations reveal that the synergistic interaction between surfactants and compound acids enhances water molecule diffusivity, improving coal wettability and promoting pore and fracture network development. This synergistic effect not only further reduces coal strength but also significantly enhances permeability, which is vital for mitigating stress accumulation in coal seams. This provides critical theoretical and technical guidance for the effective prevention and control of dynamic disasters such as rock bursts.

## Introduction

 Coal resources make up 94% of China’s energy resources. Due to China’s abundance of coal and lack of oil and gas, coal has become the foundation of the country’s rapid development^1–3^. As shallow coal strata are depleted, mining operations are moving to deeper strata^4–6^. However, extracting coal from deep strata presents challenges due to high geo-stress and complex geological structures, resulting in difficulties in preventing rockbursts and gas disasters, and the dust suppression effect of coal seam water injection is increasingly compromised.

In a study conducted by Ali et al^7^., uniaxial compression tests were performed on coal samples, with the resulting acoustic emission signals being monitored. These signals were then subjected to further analysis using fractal theory, with the results indicating a significant reduction in the mechanical properties of saturated coal samples, including uniaxial compressive strength (UCS), dissipated energy, peak stress, and modulus of elasticity. Feng et al^8^. investigated the extension of cracks in coal during the damage process, as well as the patterns and response characteristics of acoustic emission (AE) and electromagnetic radiation (EMR). While these studies have advanced understanding of coal’s mechanical response under stress or corrosion, they primarily focus on single-factor effects (e.g., water saturation, acid alone) and lack integration of macro-mechanical behavior with microscopic changes. The development of crack extension in the porous coal body is evident at the peak loading stage, leading to intensified damage damage, and fracture of the coal. Zhou and Xu et al.^9,10^conducted uniaxial compression tests on vertical-stratified coal samples (CSVB) and parallel-stratified coal samples (CSPB) with a focus on coal samples with impact tendency. The results demonstrated a positive correlation between the loading rates and the uniaxial compressive strengths of both CSVB and CSPB. Furthermore, the acoustic emission counts and cumulative absolute acoustic emission energy of both CSVB and CSPB increased with the loading rate. Nevertheless, the acoustic emission parameters of CSVB were found to be greater than those of CSPB. Yu et al^11^. used uniaxial compression and acoustic emission monitoring to analyse the mechanical properties of granite with different corrosion times, and obtained the change rule of acoustic emission characteristic parameters, and concluded that with the increase of corrosion time, the cumulative acoustic emission ringing number decreased, and the high-level acoustic emission ringing number decreased. Zhang et al^12^. analysed the acoustic emission information and the change rule of peak frequency distribution of granite in the crack extension stage by uniaxial compression test. The microcrack type reflected by RA-AF feature was found to be more consistent with the macro fracture morphology. Tian et al^13^. analysed the infrared radiation distribution of coal. The infrared radiation temperature distribution were employed to investigate the temperature change law of coal as reflected by infrared radiation. The results indicated the existence of a temperature agglomeration phenomenon during the coal damage process. Kong et al^14^. put forth a method for identifying critical damage that combines the analysis of strain and temperature data. This method examines the change rule of the strain field and temperature field before rock damage occurs. Zhang et al^15^. conducted a study to investigate the damage evolution characteristics of different coals. A relationship between microscopic and macroscopic damage in coal was calculated based on the acoustic emission localisation scenario. Song et al^16^. prefabricated cracks with different inclination angles in coal to simulate primary cracks, and uniaxial compression experiments were carried out on these coals and rocks to investigate the mechanism of the influence of prefabricated cracks on the intensity of electromagnetic radiation.

Jin et al^17^. studied the infiltration characteristics of different coal types in pure water and dust binder solutions, and the results showed that the bedding direction and solution characteristics of coal have a significant impact on the wetting effect of coal infiltration. Wu et al^18^. conducted an experimental study on the wettability and compounding effect of surfactants and showed that most surfactants showed improved wettability after compounding. Ni et al.^19,20^added the surfactant SDS to the traditional compound acid (HCl + HF) for testing, and the results showed that the surfactant had a strong acidification synergy. Cheng et al^21^. to solve the problem of high dust concentration in coal mining face. Despite evidence that surfactants enhance acidification, few studies have systematically linked this synergy to changes in coal’s chemical structure, pore connectivity, and mechanical strength—creating a critical gap in understanding how to optimize this technology for dust suppression and rock-burst mitigation. The contact angle and surface tension of different surfactants and mixed reagents were measured. The results showed that the composite dust suppressant had good inhibition effect on respirable coal dust. Golfier. F et al^22^. developed an averaging model for predicting the spread of vermivores. Balakotaiah. V et al^23^. studied the effect of pore structure and reaction rate on mass transfer coefficient. Zhang et al^24^. proposed that in complex oil and gas reservoirs with special lithology, the acid system should evolve from a single type to a composite type, forming a multifunctional acid system with filtration reduction, velocity inhibition, corrosion inhibition, resistance reduction and drainage assistance. Li et al^25^. clarified that when the acid concentration is large, the acid concentration has no obvious effect on the acidification effect, and pointed out the importance of improving the permeability of coal by improving the acidification transformation pore and fracture system.

Li et al^26^. investigated the contact angle test of surfactants on coal dust with different coal qualities and particle sizes. The results showed that the wettability of coal increased with the increase of fractal dimension of the pore structure on the coal surface. Qin^27^ and Jia^28^used fractal theory to compare and analyse the pore characteristics of coal before and after acidification. The pore connectivity of coal increased after acidification. The specific surface area and pore fractal dimension of coal became smaller, the pore structure tended to simplify, and the coal surface became smoother. Wang et al^29^. used bituminous coal as a research object. Solvent extraction experiments were carried out on coal samples at different solvent concentrations before and after acidification. The study showed that high concentration has more obvious effect on coal acidification. Ma et al^30^. conducted an experimental study on coal samples using HCl and polyethylene (PE). It was found that the treated coal macromolecular structure was looser and the average pore size increased significantly. Wang et al^31^. conducted field tests on coal seams using HCl and HF. It was found that the porosity of the coal seam increased after acidification, and the maximum gas extraction increased to 2,400 m^3^. Turenr et al^32^. conducted core permeability testing experiments using hydrochloric acid and found that the use of hydrochloric acid to dissolve minerals increased the permeability of coal seams. Yu et al.^33–35^ conducted research into the solubilization pattern of acetic acid on functional groups and microstructure of coal samples. Their findings indicated that high concentrations of acetic acid had the most significant effect on the micro characteristics of coal. Xu et al.^36–39^ investigated the effects of acids and bases, organic and inorganic acids on the unreduced characteristics of coal. The research demonstrated that inorganic acids had the most beneficial effect on improving the strength and wettability of coal.

Recent studies have further explored acid-surfactant synergy in coal reservoirs: Li et al^40^. reported that SDS-modified organic acids increase coal permeability by 2.3 times via pore-fracture network expansion, while Zhao et al^41^. highlighted that SDS reduces acid-rock reaction time by 30% by improving acid diffusion. However, these works lack systematic integration of macro-mechanical responses (e.g., AE/EMR) with molecular-level interactions—an gap addressed by our multi-scale study.

In summary, many studies have been carried out on the mechanical characteristic and microscopic characterisation of coal samples. However, there are fewer joint studies on mechanical characteristic and microscopic characteristics based on acidification. Unlike previous single-characterization studies, this work combines mechanical testing, spectroscopic analysis, NMR, and molecular dynamics simulations to systematically investigate the synergistic effects of SDS and compound acids on coal’s mechanical and microscopic properties. FTIR was used to analyse the change law of the four functional groups of coal samples, and the influence of the influence of the chemical structure of composite acidification-treated coal on wettability was clarified. At the same time, the micro-mechanism of the effect on the wettability of coal was explored with molecular dynamics simulation. Then, NMR technology was used to comprehensively analyse the pore structure characteristics of the experimental coal samples and to determine the damage law of the microscopic pore structure of the compound acidified water-injected coal. Finally, fractal theory was employed to quantitatively investigate the evolution law of coal microporous structural features and to further evaluate the fractal dimension characteristics of coal micropores following acidification.

## Materials and methods

**Fig. 1:**
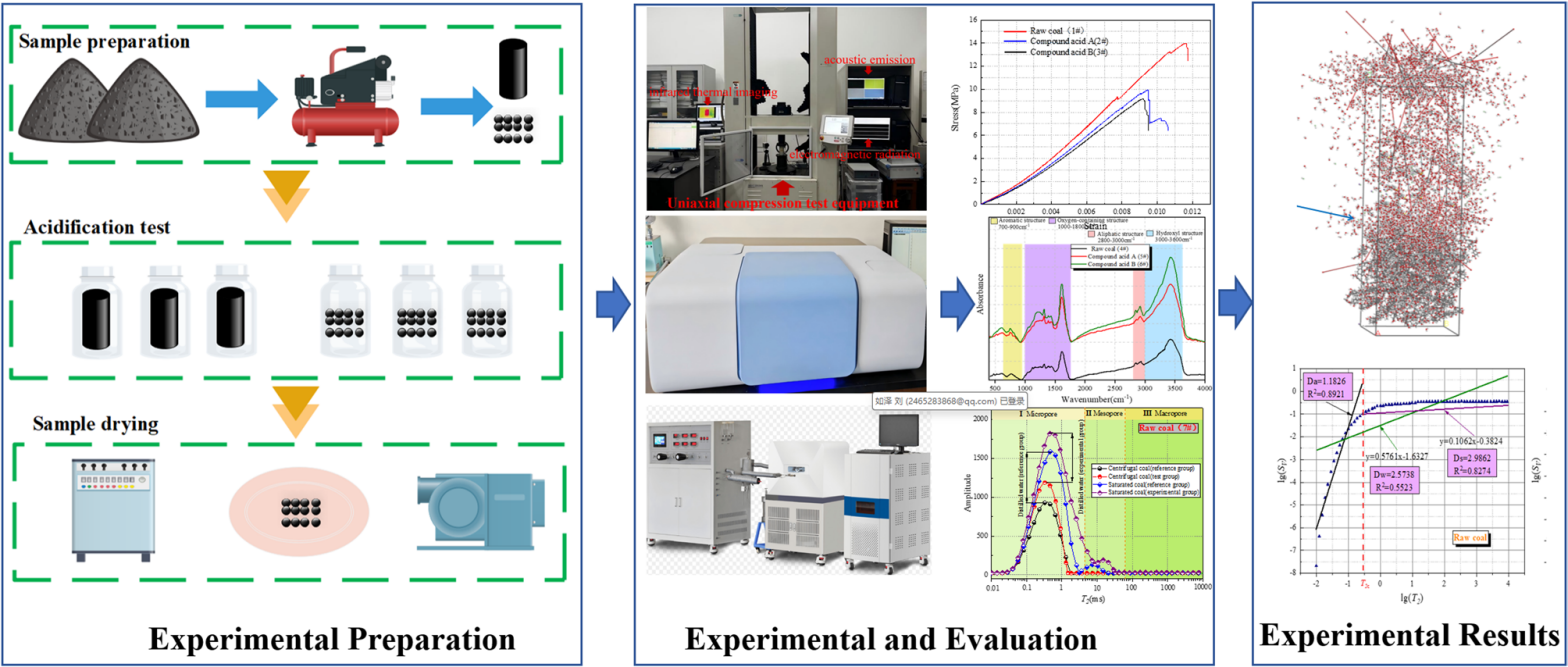
Schematic of the experimental process.

To address this gap, we integrate multi-scale characterization (macro-mechanical testing, micro-spectroscopy, and molecular simulations) to investigate how sodium dodecyl sulfate (SDS) and compound acids synergistically modify coal’s properties. The overall experimental workflow of this study is schematically illustrated in Fig. 1, including coal sample preparation and chemical treatment, mechanical and microscopic testing procedures, as well as the molecular dynamics simulation framework used to interpret the experimental results.

### Sample preparation

The coal samples were obtained from a low-permeability coal seam. Coal blocks were collected from the working face, sealed on site, and transported to the laboratory. All specimens were prepared from the same bulk coal to avoid variability among samples. Homogeneity was verified using ultrasonic velocity measurements Table 1.

A total of 9 coal samples (1#~9#) were prepared, with 3 replicates per treatment condition: 1#~3# for uniaxial compression tests, 4#~6# for FTIR analysis, and 7#~9# for NMR tests. All samples were cored from the same bulk coal (diameter = 50 mm, height = 100 mm) and validated for homogeneity via ultrasonic velocimetry (velocity variation < 5%).

**Table 1:** Fundamental properties of the coal.

Property category	Index	Value	Testing method	Testing method
Coal rank	Type	Gas coal	GB/T 5751 − 2009 (Chinese National Standard for Coal Classification)	GB/T 5751 − 2009 (Chinese National Standard for Coal Classification)
Proximate analysis	Moisture (Mₐᵈ)	2.31% ± 0.08%	GB/T 211–2017 (Determination of Moisture in Coal)	GB/T 211–2017 (Determination of Moisture in Coal)
	Ash content (Aᵈ)	9.45% ± 0.12%	GB/T 212–2008 (Proximate Analysis of Coal)	GB/T 212–2008 (Proximate Analysis of Coal)
	Volatile matter (Vᵈᵃᶠ)	38.62% ± 0.21%	GB/T 212–2008	GB/T 212–2008
Maceral analysis	Vitrinite reflectance (R_r_ₘₐₓ)	0.82% ± 0.03%	GB/T 6948 − 2018 (Determination of Vitrinite Reflectance of Coal)	GB/T 6948 − 2018 (Determination of Vitrinite Reflectance of Coal)
Mineral composition	Calcite (CaCO₃)	4.2%	X-ray diffraction (XRD, Bruker D8 Advance)	X-ray diffraction (XRD, Bruker D8 Advance)
	Pyrite (FeS₂)	2.8%	XRD	XRD
	Clay minerals (illite)	3.1%	XRD	XRD
Physical property	Initial permeability	0.08 mD ± 0.01 mD	Steady-state gas permeability test	Steady-state gas permeability test

The coal sample was collected from the No. 3 coal seam of Guotun Coal Mine (Shandong, China), a typical low-permeability gas coal seam. Fundamental properties were characterized via standard methods: proximate analysis to determine moisture, ash, and volatile matter; vitrinite reflectance testing to confirm coal rank; and XRD to identify mineral composition (calcite, pyrite, and clay minerals are the main acid-reactive components). The initial permeability (0.08 mD) is consistent with the low-permeability coal seam context stated in the study, ensuring the sample’s representativeness for deep mining scenarios.

The coal samples were immersed in three solutions: distilled water (A), compound acid (B: 3% HCl + 3% CH₃COOH + 3% HF + 2% KCl), and SDS-modified compound acid (C: B + 5% SDS). Samples 1#–9# correspond to these three treatment groups. The soaking duration was 72 h.

Figure 1 illustrates the experimental workflow, including sample preparation and treatment, experimental equipment and testing procedures, and the acquisition of mechanical and microscopic results. It also outlines the molecular dynamics simulation framework used to interpret the macroscopic experimental findings from a microscopic perspective.

### Uniaxial compression test

**Fig. 2:**
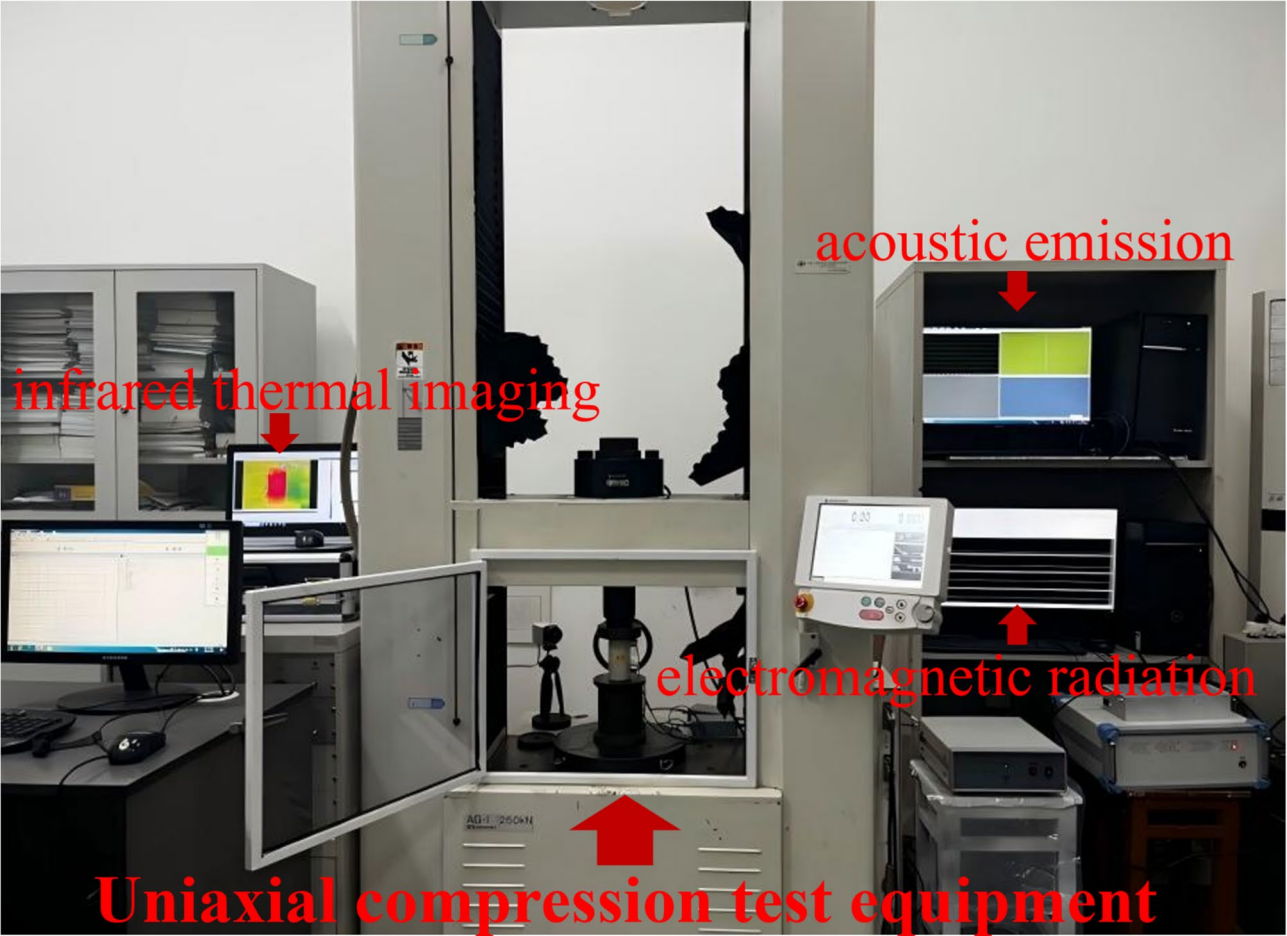
Uniaxial compression testing system.

Uniaxial compression testing is a standard approach in rock mechanics to assess deformation and mechanical behavior. The experimental setup is shown in Fig. 2 and includes acoustic emission, electromagnetic radiation, and infrared thermal imaging modules.

Uniaxial compression tests were conducted in accordance with the Chinese national standard GB/T 23561.1–2009, 《Determination of Mechanical Properties of Coal and Rock—Part 1: Uniaxial Compression Test》. A servo-controlled testing machine (MTS 815) was employed, applying a constant displacement rate of 0.5 mm/min, in accordance with typical coal mechanics protocols^7,11^, until specimen failure was observed. The testing system was equipped with acoustic emission (AE: RAE-2 A), electromagnetic radiation (EMR: EMR-200), and infrared thermal imaging (FLIR A655sc) modules to simultaneously monitor energy release and temperature variations.

### Fractal dimension theory

Mandelbrot introduced fractals and fractal theory in 1975, and these concepts have since been widely applied across scientific fields, including the characterization of natural materials^42,43^. For coal, fractal dimension theory has proven effective in assessing micro-pore structures due to the geometric complexity inherent to coal reservoirs^44^.

In this study, fractal dimension theory was used to characterize pore structures based on NMR *T*_*2*_ spectra following Song et al. Three fractal dimensions were defined:

*D*_*W*_ (total pore fractal dimension): Calculated using the full *T*_*2*_ range (0.01–10,000 ms), representing overall pore complexity. *D*_*S*_ (seepage pore fractal dimension): Derived from *T*_*2*_ > 10 ms, corresponding to free water in meso- and macropores. *D*_*a*_ (adsorption pore fractal dimension): Derived from *T*_*2*_ < 10 ms, characterizing micropores containing bound water.

The fractal dimension *D* was obtained from the linear relationship between lg(*S*_*V*_) (specific surface area) and lg(*T*_*2*_) (transverse relaxation time), with the formula:

1 $$D=3 - \frac{{\lg ({S_2})}}{{\lg ({T_2}) - \lg ({T_{2max}})}}$$

where *T*_*2max*_ is the maximum *T*_*2*_ value of the sample. This formula is further elaborated in Sect. 4.1 with NMR-specific data.

### Molecular dynamics simulations

**Fig. 3:**
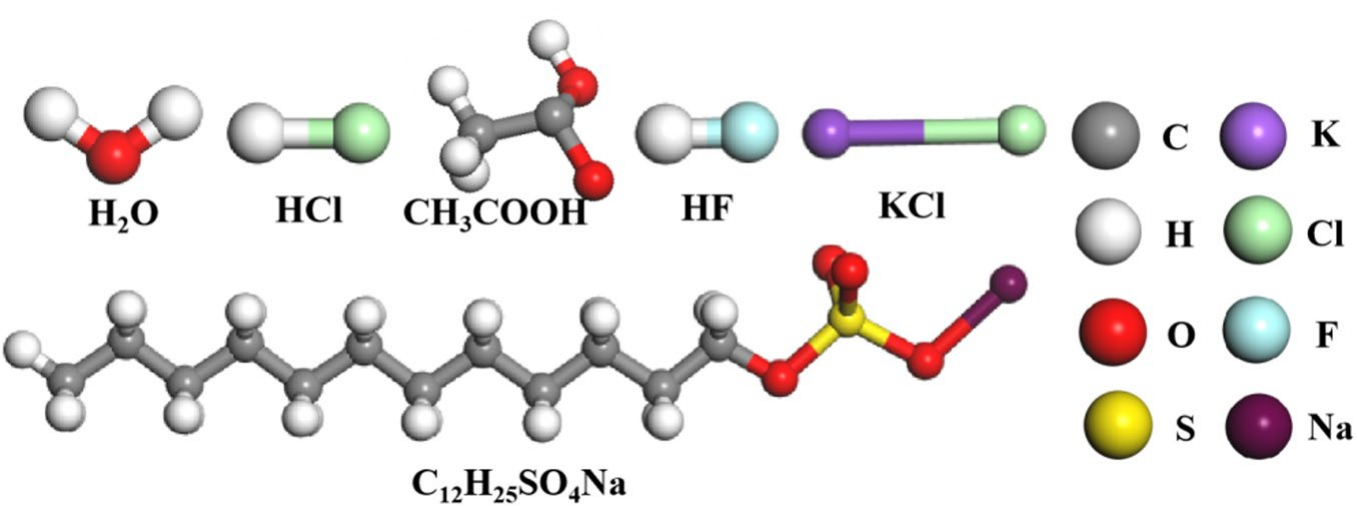
Molecular structure model.

Composite acidic solution C (3% HCl + 3% CH₃COOH + 3% HF + 2% KCl + 5% SDS) was selected for molecular simulations. The constructed molecular structure model is shown in Fig. 3.

**Fig. 4:**
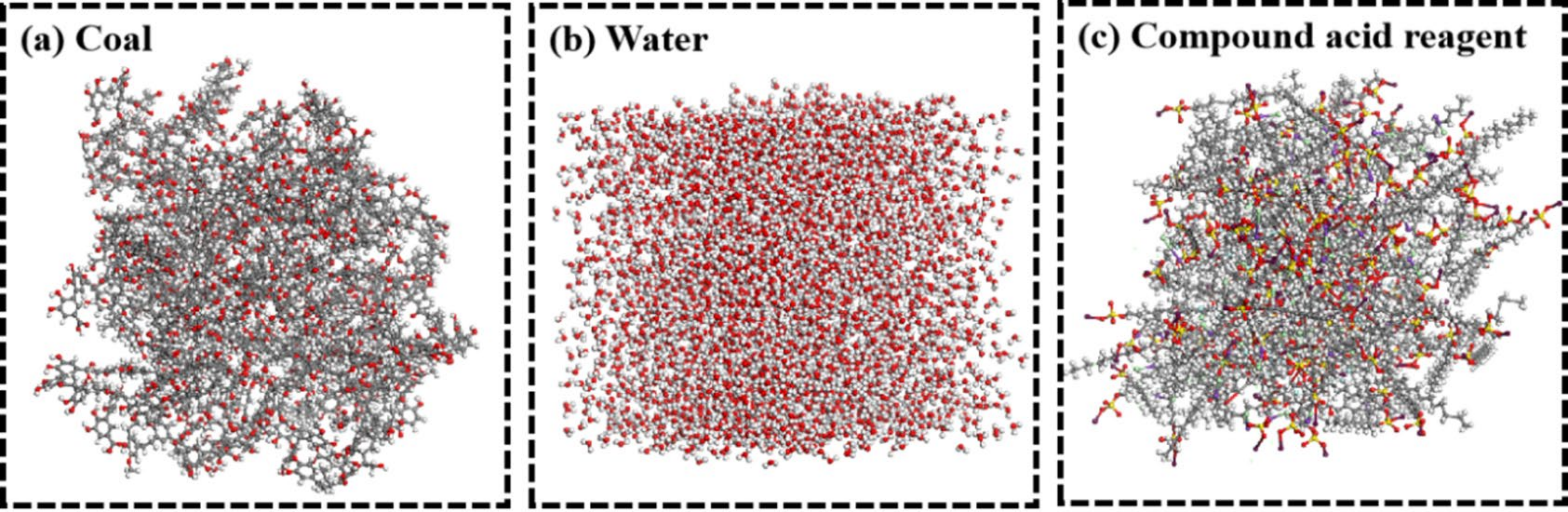
Molecular system model.

A molecular system consisting of coal, water, and the composite acid solution was established to investigate the wetting and spreading behavior of the acid solution on the coal surface. This model, shown in Fig. 4, enabled exploration of the interaction mechanisms between the composite acid and coal at the molecular scale.

To analyze the dynamic behavior of water molecules within the composite acid system, the mean square displacement (MSD) and diffusion coefficient (D) were calculated:

2 $$\:\mathrm{M}\mathrm{S}\mathrm{D}=\frac{1}{\mathrm{N}}\sum\:_{\mathrm{i}=1}^{\mathrm{N}}{\left[{\mathrm{r}}_{\mathrm{i}}\left(\mathrm{t}\right)-{\mathrm{r}}_{\mathrm{i}}\left(0\right)\right]}^{2}$$

3 $$\:\mathrm{D}=\frac{1}{6\mathrm{N}}\underset{\mathrm{x}\to\:{\infty\:}}{\mathrm{lim}}\frac{\mathrm{d}}{\mathrm{d}\mathrm{t}}\sum\:_{\mathrm{i}=1}^{\mathrm{N}}{\left[{\mathrm{r}}_{\mathrm{i}}\left(\mathrm{t}\right)-{\mathrm{r}}_{\mathrm{i}}\left(0\right)\right]}^{2}=\frac{1}{6}{\mathrm{K}}_{\mathrm{M}\mathrm{S}\mathrm{D}}$$

where *N* is the number of diffusing molecules and *r(t)* is molecular position at time *t*. These metrics provide quantitative insight into molecular transport behavior.

## Analysis of experimental results

### Experimental analysis of uniaxial compression test

**Fig. 5:**
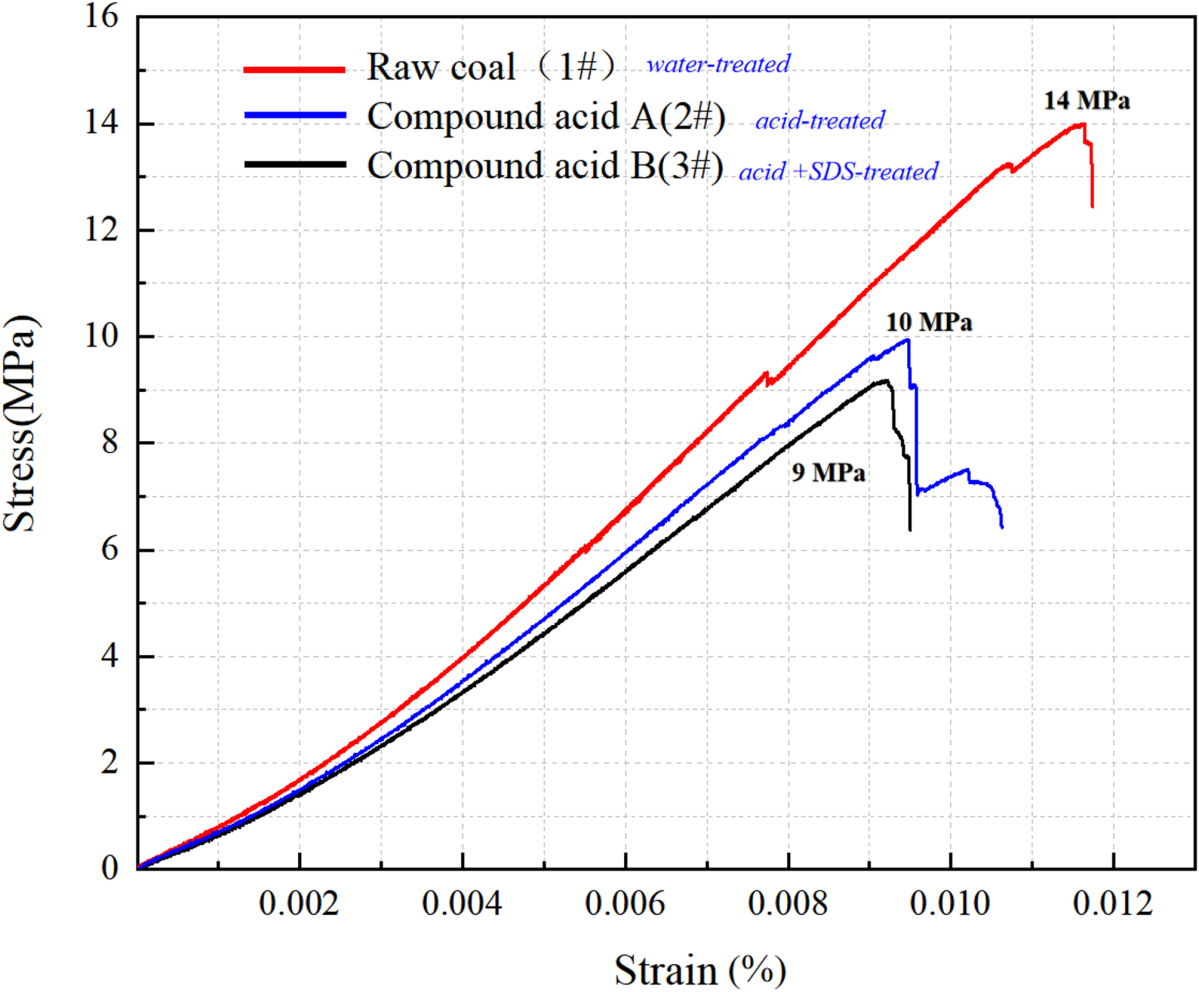
Uniaxial compression test result.

The compressive strength of the three specimen groups was analyzed. The water-soaked coal samples exhibited higher strength than the acid-treated samples, with a 30% higher peak strength and greater strain. A comparison of the two acid-treated samples showed that sample 2# had slightly higher strength than the SDS-treated sample 3#, with an increase of approximately 10%. The results indicate that acidification decreases coal’s uniaxial compressive strength, and the addition of SDS further accelerates pore and fissure development in acid-corroded coal, thereby reducing its compressive capacity. All strength values reported are averages of three replicate samples (1#: water-treated, 2#: compound acid-treated, 3#: compound acid + SDS-treated), with standard deviations (±) indicating variability. The stress–strain responses of coal samples under different treatment conditions (Fig. 5) demonstrate that acidification significantly reduces uniaxial compressive strength, and this weakening effect is further enhanced by the addition of SDS Table 2.

**Table 2:** Uniaxial compressive strength (UCS) of coal Samples.

Sample	Treatment condition	UCS (MPa) ± SD	Strength reduction vs. 1#	Strength reduction vs. 2#
1#	Distilled water	18.2 ± 0.5	—	—
2#	Compound acid (no SDS)	12.7 ± 0.3	30.2%	—
3#	Compound acid + 5% SDS	11.4 ± 0.4	37.4%	10.2%

The UCS of water-treated samples (1#) was 18.2 ± 0.5 MPa, whereas acid-treated samples (2#) showed a 30.2% reduction to 12.7 ± 0.3 MPa. Adding SDS (3#) further reduced UCS by 10.2% to 11.4 ± 0.4 MPa, confirming the synergistic weakening effect of SDS.

**Fig. 6:**
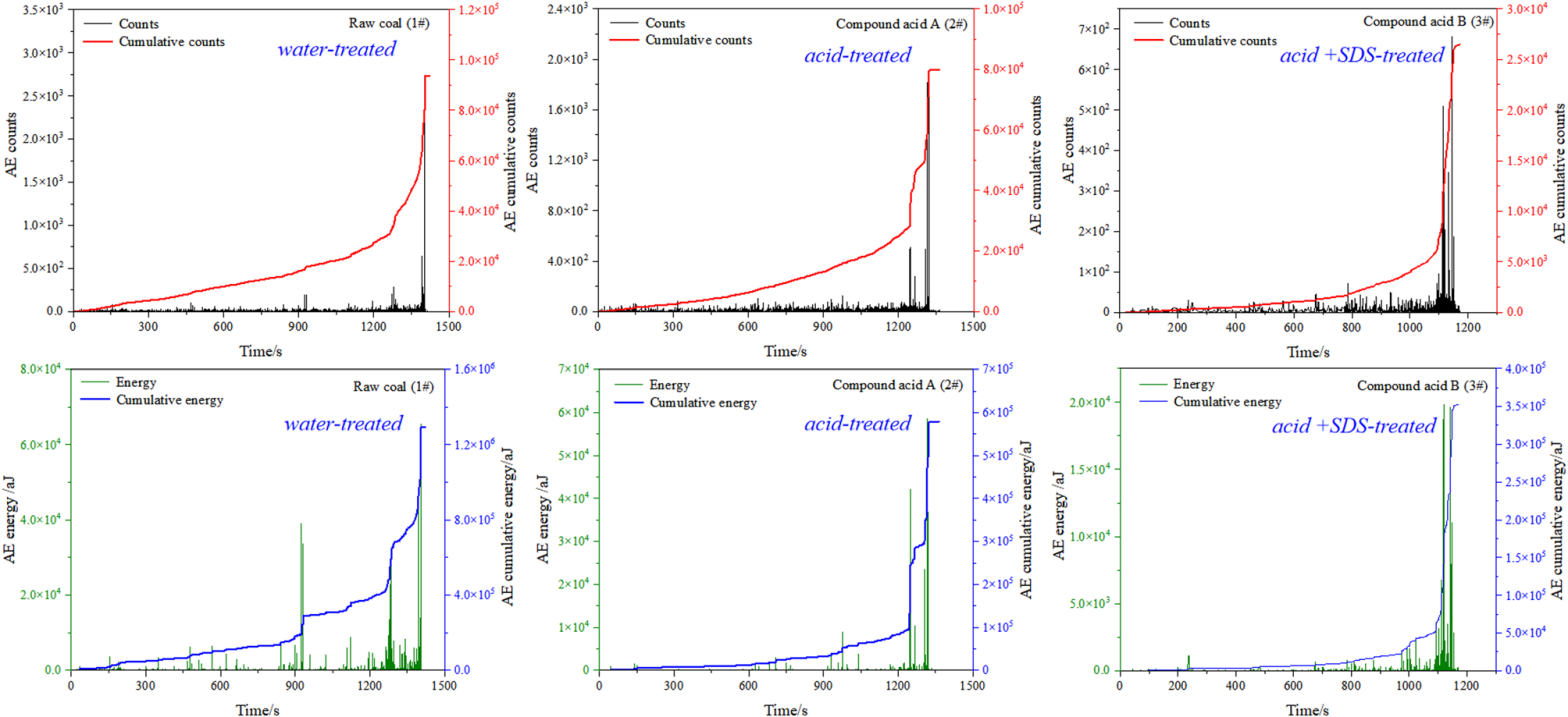
AE counts and energy.

As shown in Fig. 6, both the cumulative acoustic emission (AE) counts and energy decrease markedly after acid and SDS-assisted acid treatments, indicating a reduction in stress concentration during coal failure. The acoustic emission (AE) count and energy data were also analyzed. Water-treated samples exhibited larger AE energy and cumulative energy values. Both acid-treated groups showed lower energy, but differences existed between them. Samples treated with SDS displayed relatively lower energy values, with peak energy being about 6% lower than sample 2#, indicating that SDS promotes acidification and weakens the coal, resulting in reduced energy release.

**Fig. 7:**
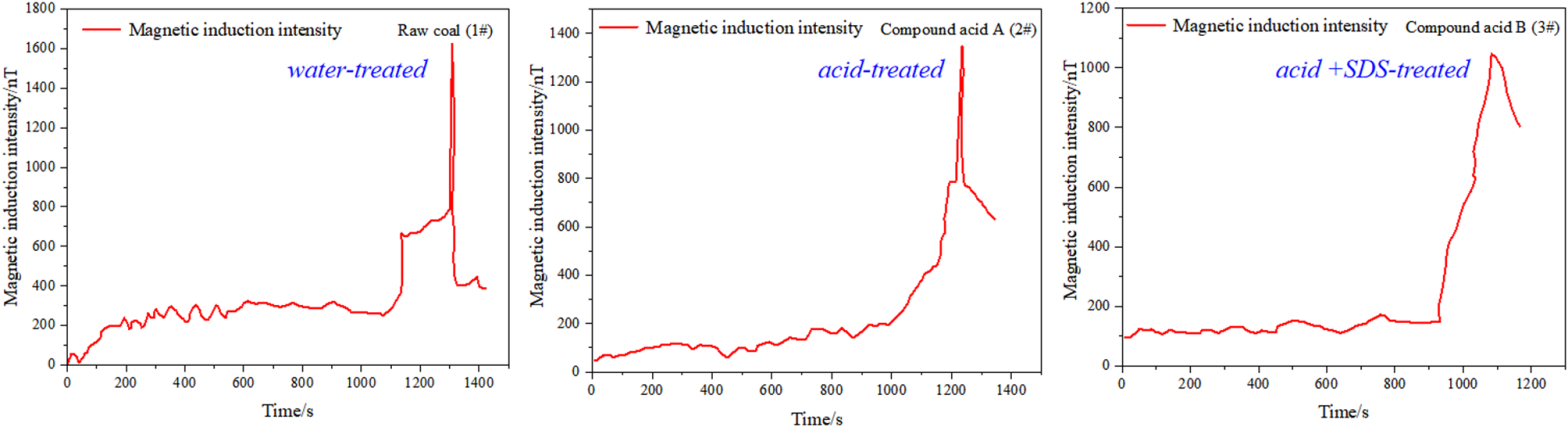
Electromagnetic radiation intensity.

The electromagnetic radiation (EMR) responses recorded during the loading process are presented in Fig. 7, revealing a noticeable attenuation of EMR intensity for chemically treated coal samples. Electromagnetic radiation (EMR) monitoring reflects variations in the electric field generated by frictional charge transfer during coal failure. It also provides information on the intensity of energy release. A comparison of EMR intensity among the samples is shown below.

Analysis of the EMR intensity revealed that sample 1# exhibited the highest peak intensity, followed by 2# and 3#. The EMR energy pattern closely resembled the AE energy results.

**Fig. 8:**
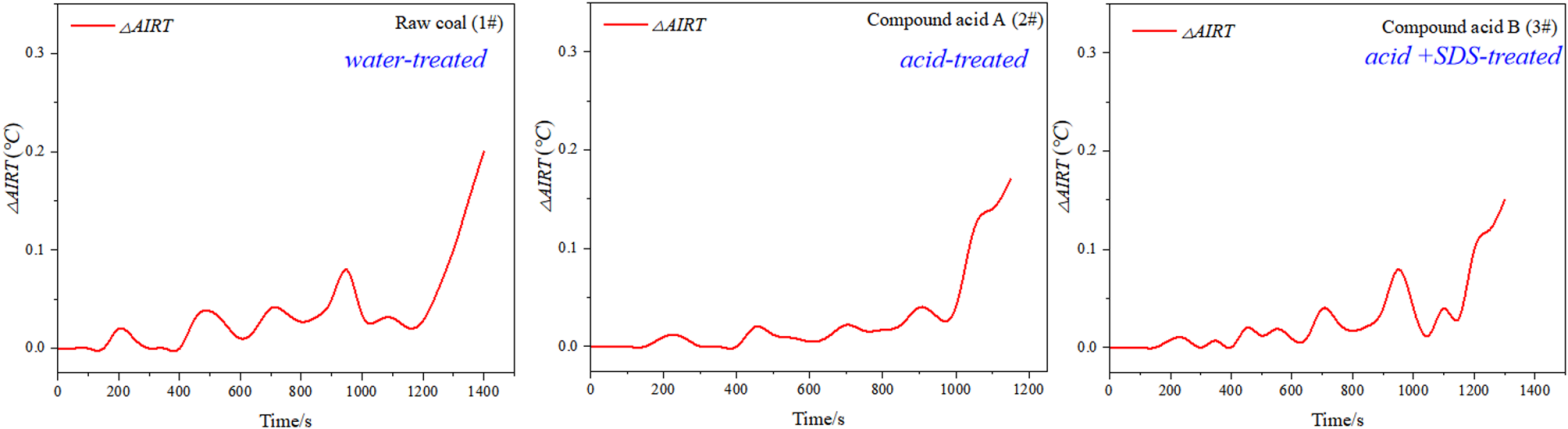
Infrared radiation temperature variation curve.

The variations in infrared radiation temperature during uniaxial compression are shown in Fig. 8, reflecting differences in energy accumulation and dissipation behaviors among the treated and untreated coal samples. Coal sample 1# showed the largest increase in infrared radiation temperature (approximately 0.2 °C), while samples 2# and 3# both increased by around 0.15 °C. Acidification reduced the coal samples’ ability to accumulate energy, resulting in a lower released energy value.

SDS enhances acid-induced coal weakening via two interrelated mechanisms: (1) Chemical modification (FTIR, Sect. 3.2): SDS reduces aromatic content by ~ 30% and aliphatic content by ~ 50%, breaking coal’s hydrophobic macromolecular framework and exposing more reactive sites for acid attack. (2) Microstructural evolution (NMR, Sect. 3.3): SDS increases acid diffusion into pores, dissolving impurity minerals (e.g., calcite, pyrite) that block fractures. This raises porosity by 14.5% (vs. 8.2% for acid alone) and improves pore connectivity (lower *D*_*W*_ and *D*_*S*_), reducing coal’s ability to resist compressive stress.

The lower AE/EMR energy of acid-SDS treated coal (3#) stems from reduced stress concentration: NMR data show interconnected pores/fractures (from SDS-aided acidification) dissipate stress gradually, rather than accumulating to trigger sudden energy bursts (as in raw coal, 1#). This is consistent with infrared data (Fig. 8), where 3# shows a smaller temperature increase (0.15 °C vs. 0.2 °C for 1#), indicating less localized energy release.

In summary, this experiment measured uniaxial strength along with changes in infrared radiation temperature, AE energy, and EMR intensity. The results showed that acidification-treated sample had a significant effect on the strength of the coal. Specifically, the strength of the 1#, 2#, and 3# coal samples gradually decreased, which was also reflected in the corresponding emission and electromagnetic radiation energy. The infrared radiation temperature increase of coal sample 1# is the most pronounced and coal sample 2# and coal sample 3# are basically the same. The overall effect of acidification is to reduce the strength of the coal, while the addition of SDS promotes acidification and effectively reduces the strength of the coal.

### Experimental analysis of FTIR

**Fig. 9:**
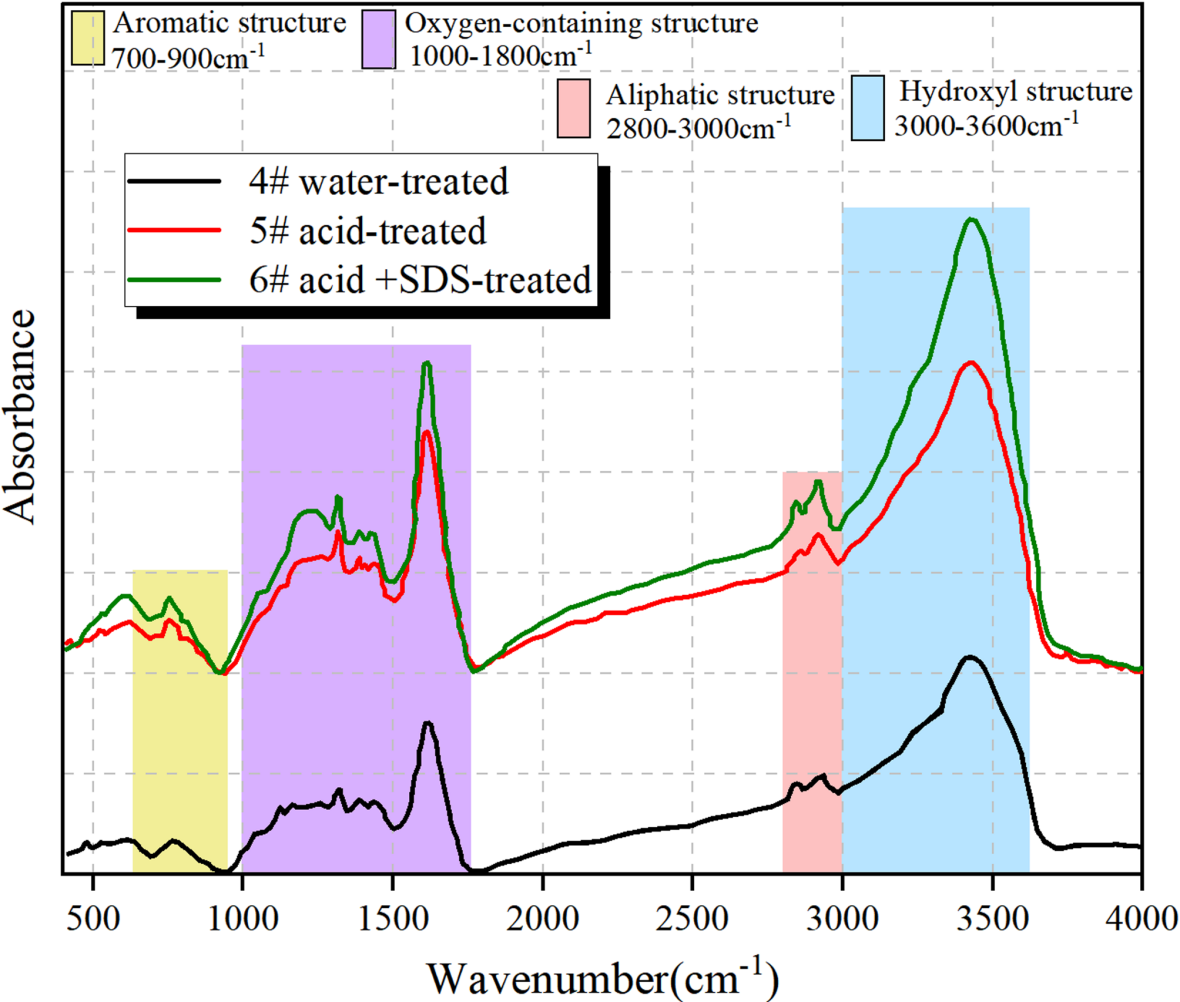
FTIR spectral line of coal samples.

The absorption probability of a particular infrared frequency is determined by both the frequency and the interaction between molecules. This information is crucial in determining the molecular structure of coal, including its chemical functional groups. The experimental coal samples were tested, and their infrared spectra were obtained. Figure 9 shows the baseline-corrected infrared spectrum. FTIR results represent averages of 3 replicate samples per group, with peak area < 4% confirming reproducibility.

Figure 9 shows that the treated coal samples exhibit comparable characteristics. It demonstrates that the surface structure and functional groups of the treated coal samples are identical to those of the original coal. The discrepancies are primarily reflected in the distribution characteristics and content of functional groups. The infrared spectral curves of the coal samples can be analysed by curve fitting to determine the distribution and content parameters of different chemical functional groups. In order to facilitate quantitative analysis of the functional groups present in the coal samples, the FTIR spectral data was divided into four distinct absorption bands: aromatic structure (700–900 cm^− 1^), oxygenated structure (1000–1800 cm^− 1^), aliphatic structure (2800–3000 cm^− 1^) and hydroxyl structure (3000–3600 cm^− 1^). Due to the possibility of multiple bands within a single absorption peak, resulting in overlap between neighboring peaks, direct measurement of peak height and area is not an accurate representation. To accurately measure peak height and area, the absorption peak curve must be fitted.

Semi-quantitative FTIR data confirm SDS’s role in modifying coal chemistry: Aromatic and aliphatic contents decrease by ~ 30% and ~ 50% (vs. raw coal), respectively, due to SDS-facilitated acid cleavage of C-C bonds in hydrophobic structures. Oxygenated groups (C-O, C = O) increase by ~ 13% (6# vs. 4#), enhancing hydrophilicity—consistent with improved coal wettability observed in molecular dynamics simulations.

**Fig. 10:**
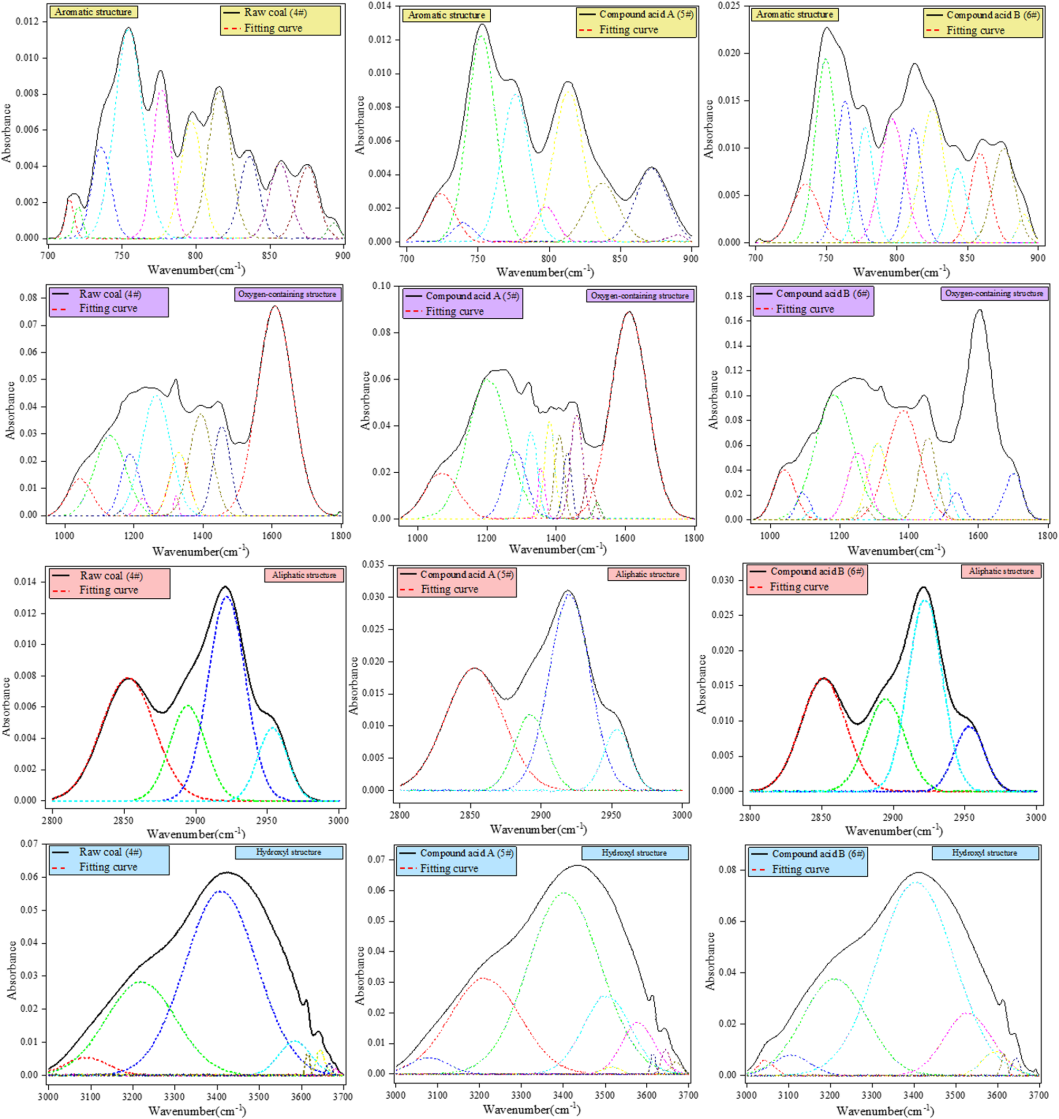
Peak-fitting curves of different structures.

The peak-fitting results of functional groups derived from FTIR spectra (Fig. 10) indicate a pronounced reduction in aromatic and aliphatic structures after SDS-assisted acidification. The acidification-treated sample has an impact on the functional groups present in coal. The specific effects of this treatment can be analysed by calculating the peak areas of various functional groups. The results indicate that the 700–900 cm^− 1^ band corresponds to the out-of-plane deformation vibrations of aromatic hydrogen atoms in coal. This band is indicative of the varying degrees of substitution observed in the aromatic structures. The results of the substitution of aromatic structures indicate that the addition of SDS promotes the substitution effect.

The band between 1000 and 1800 cm^− 1^ is associated with oxygen-containing structural groups in coal. The acidic compound solution, with the SDS acidification, has a greater impact on disrupting the bridge bonds that connect inorganic elements to oxygen-containing functional groups. The relative content of -CH_3_ and -CH_2_ shows less fluctuation after treatment within the 1000–1800 cm^− 1^ range. The surfactant SDS promotes the conversion of various groups into C-O and C = O groups during compound acidification.

The aliphatic structures in coal are primarily distributed within the range of 2800–3000 cm^− 1^. The infrared spectra of the coal samples exhibited comparable curves. The peak shapes of the aliphatic structures remained essentially unaltered following acidification-treated sample. The hydroxyl group is the primary source of hydrogen bonding in coal, and its absorption vibration peaks are located within the range of 3000–3600 cm^− 1^. The acidification process results in the disruption of the weak chemical bonds between hydrogen atoms. The addition of SDS has a significant effect on the disruption of coal bridge bonds.

### Experimental analysis of NMR

The pore structure of coal rock can be divided into three categories: microporous (pore size < 10 nm), mesopores (10 nm < pore size < 100 nm), and macropores (100 nm < pore size < 105 nm)^45,46^. The samples were dried at 65 °C, weighed to record the mass after drying, and the *T*_*2*_ spectrum of the dried samples was measured. The samples were then evacuated for 6 h and saturated with water for 12 h to measure the *T*_*2*_ spectrum. The samples were centrifuged for 20 min using a high-speed centrifuge to remove the internal liquid, and the *T*_*2*_ spectra were measured. NMR results represent averages of 3 replicate samples per group, with porosity variations < 4% confirming reproducibility.

Table 3 presents the porosity of coal samples before and after treatment under different conditions, porosity was calculated via inversion of low-field NMR T₂ elaxation signals. The porosity of the coal samples increased under different treatment conditions, with increases of 1.3%, 8.2%, and 14.5% for 7#, 8#, and 9# coal samples, respectively. The most significant increase was observed in the 9# coal sample after SDS synergistic acidification, with a porosity increase of 6.3% compared to the 8# coal sample treated with compound acid solution without SDS. This suggests that SDS can enhance the dissolving effect of compound acid on coal samples.

**Fig. 11:**
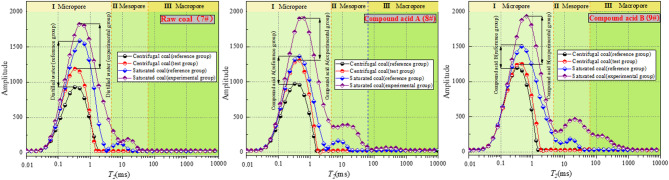
*T*_*2*_ spectrum.

The porosity increase of each sample highlights SDS’s synergistic effect: (1) Raw coal (7#) shows a negligible 1.3% increase (due to natural moisture absorption), (2) acid-only treatment (8#) raises porosity by 8.2% via mineral dissolution, and (3) acid-SDS treatment (9#) achieves a 14.5% increase—6.3% higher than 8#. This significant amplification confirms that SDS enhances acid’s ability to dissolve pore-blocking minerals (e.g., clay, pyrite) and expand fracture networks, as visualized in NMR T₂ spectra (Fig. 11: stronger peaks II/III for 9#).

**Table 3:** Porosity data.

Sample ID	Treatment condition	Porosity before treatment(%)	Porosity after treatment(%)
7#	Raw coal (untreated)	6.75	6.88
8#	Compound acid (without SDS)	6.79	7.61
9#	Compound acid + 5% SDS	6.87	8.32

The NMR technique is employed to assess the relaxation properties of hydrogen-containing fluids within the internal pores of coal. The inverse relaxation time (*T*_*2*_) distribution plot illustrates the *T*_*2*_ spectrum of a saturated coal sample. As illustrated in Fig. 11, the spectrum reflects the distribution of water within the three coal samples. The boundaries between bound water, intermediate water, and free water were defined using the critical T₂ value (T₂C = 10 ms), a widely accepted threshold in coal NMR research^47,48^. Specifically: T₂ < 10 ms (adsorption pores, bound water), 10 ms < T₂ < 100 ms (mesopores, intermediate water), and T₂ > 100 ms (macropores/fractures, free water).

The *T*_*2*_ curve for a centrifugally dried coal sample represents the volume occupied by the bound water in the coal, while the *T*_*2*_ curve for a saturated water sample represents the total volume occupied by water in the coal. The volume of free water is obtained by subtracting the volume of bound water from the volume of the saturated water sample. This volume represents the space occupied by the pore fractures in the coal sample.

Figure 11 illustrates that *T*_*2*_ curves for saturated water and centrifugally dried coal samples. The transverse relaxation time *T*_*2*_ is divided into three relaxation peaks (I, II, and III) within the decay range. These peaks are defined as micro-porous, medium-porous, large-porous, and fractured. The study found that the amplitude and width of the *T*_*2*_ curves of saturated coal samples were correlated with the amount of acidic solution component in the experimental treatment solution. Additionally, the size and number of pores in coal increased with the increase of acidic solution component.

Figure 11 shows that the three relaxation peaks of saturated water in coal sample 9# are enhanced. The pore diameter of coal samples treated with SDS co-acidification increased, and the pore connectivity effect was improved. The enhancement of acid etching resulted in an increase in the number of internal pores in the coal samples. In addition, some fissures filled with impurity minerals were found, and the pore body was a fissure network combined with many small fissures^49^.

The relaxation peaks of II and III are indicative of the mesoporous structure, macropores and fracture network within the coal. The results demonstrated that the porosity, macropores and fractures of the coal samples were enhanced by the SDS co-acidification-treated sample, resulting in the formation of a more intricate fracture network. SDS augmented the reaction area of the acid solution with the impurity minerals present within the pore fractures of the coal samples. This resulted in the removal of impurity minerals that would have filled the pore cracks, thereby creating a more complex pore-fracture system.

**Fig. 12:**
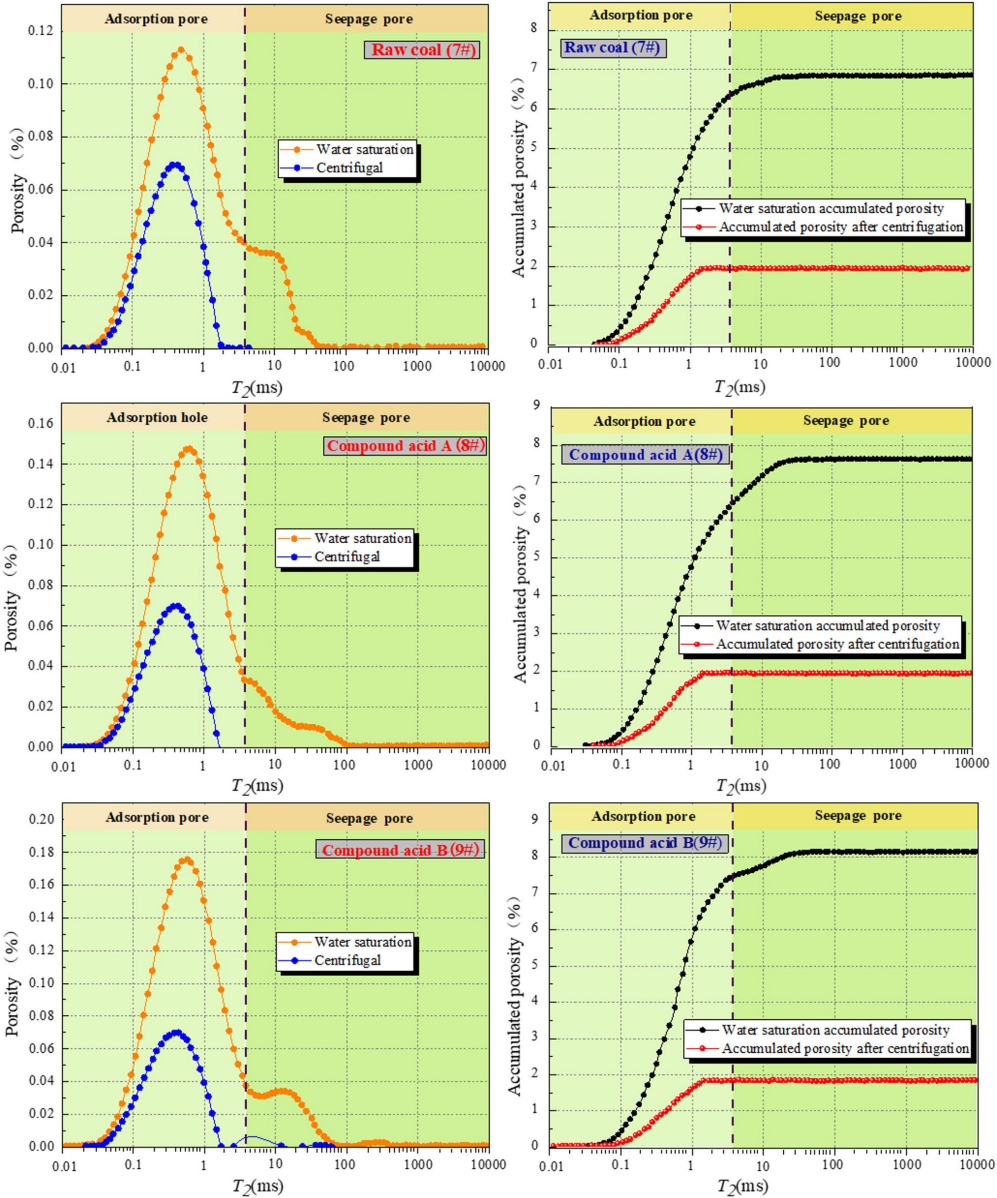
NMR *T*_*2*_ and porosity curve.

The NMR *T₂* distribution and cumulative porosity curves presented in Fig. 12 confirm that SDS-assisted acidification significantly enhances mesopore and macropore development in coal. Acidification enhanced the three relaxation peaks and increased porosity. The porosity increase in sample 9# was most pronounced. With stronger acid–SDS synergy, the T₂ peak became smoother, indicating dissolution of pore walls and accelerated crack extension.

The cumulative porosity curve allows for the calculation of the volume percentage of total pores based on their diameter sizes. The relative seepage pore space proportion decreased, possibly due to the compound acid treatment’s impact on the expansion of large pores. The effect on adsorption pores was minimal due to the acidification intensity, resulting in insignificant changes in adsorption pore volume. The cumulative porosity curve of the 9# coal sample demonstrated that the relative porosity exhibited an increase following the SDS compound acidification-treated sample in comparison to the original coal sample. In particular, the value is 6.3% higher than that of coal sample 8#. This suggests that SDS is beneficial in increasing the porosity of coal samples. Furthermore, the proportion of water-permeable pores to the total pore volume increased by 5.99%, which indicates that the addition of SDS is beneficial to the water permeability of coal.

### Microanalysis of mechanism

**Fig. 13:**
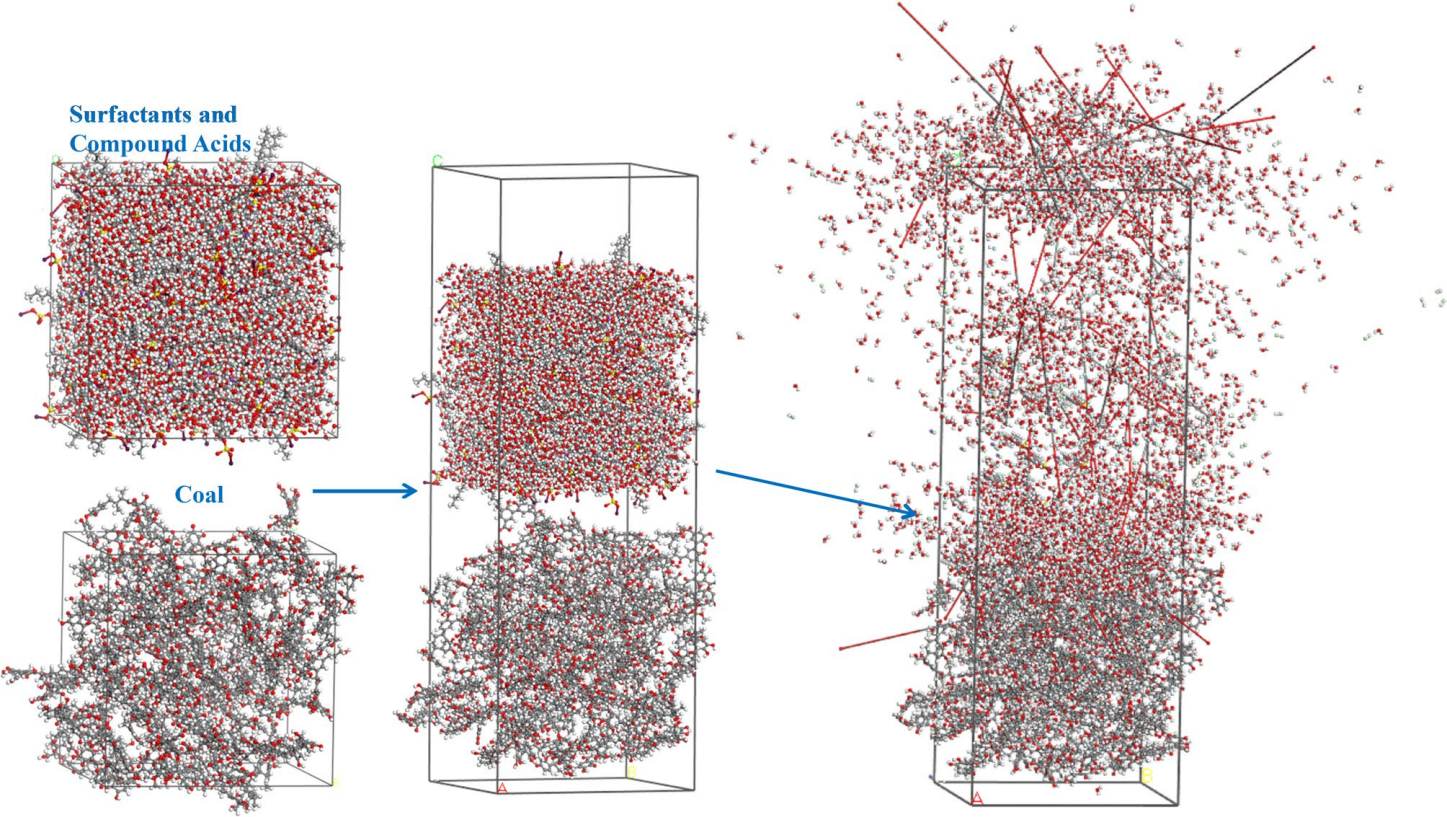
Mechanism of interaction of surfactants, compound acids and lignite.

The microscopic interaction mechanism among surfactants, compound acids, and coal is schematically illustrated in Fig. 13. During acidification, mineral components such as carbonates and clay minerals are dissolved, leading to the expansion of existing pores and the generation of new pore spaces. The addition of sodium dodecyl sulfate reduces interfacial tension and enhances the penetration of acidic solutions into the coal matrix, thereby promoting more extensive pore–fracture development. These coupled effects provide a mechanistic explanation for the observed mechanical weakening and pore structure evolution.

**Fig. 14:**
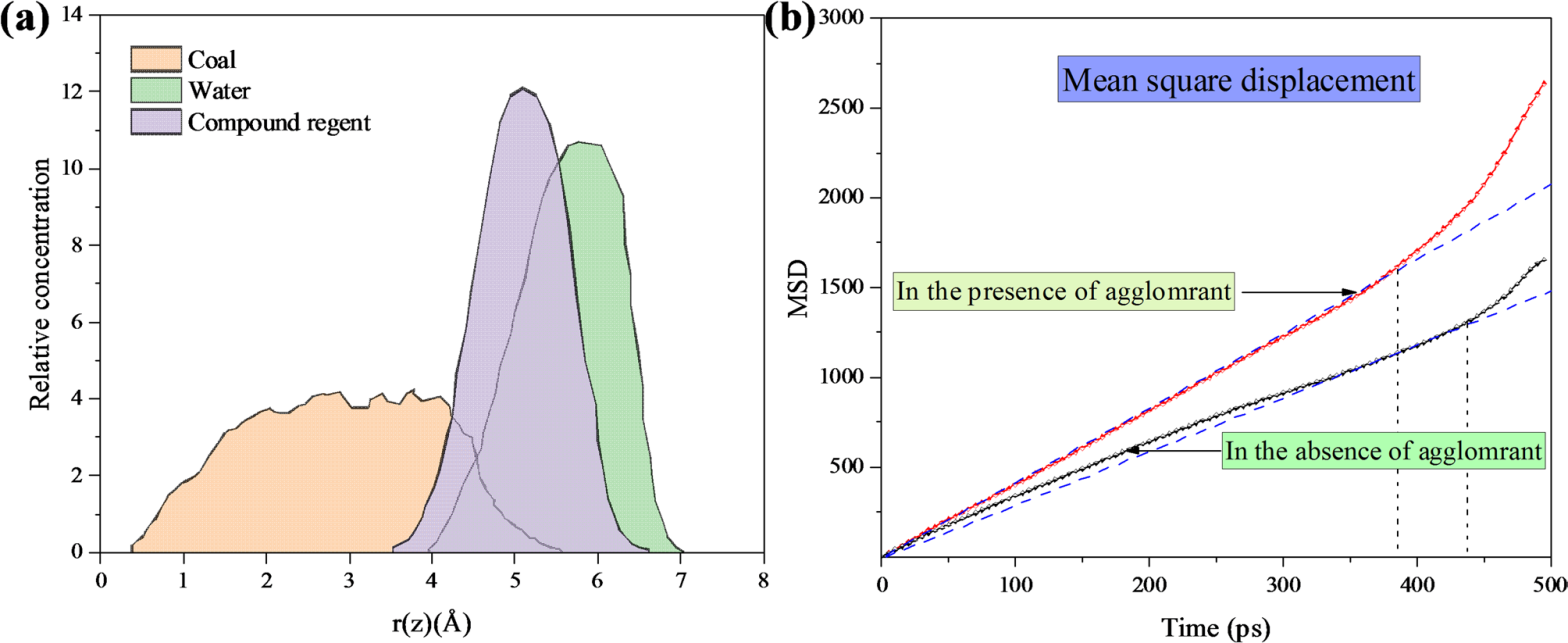
(**a**) Mass density distribution curves of water and compound reagent in the z-axis direction (**b**) MSD of water molecules.

The mass density distribution along the z-axis and the mean square displacement (MSD) of water molecules obtained from molecular dynamics simulations are presented in Fig. 14. Molecular dynamics simulation results indicate that the synergistic effect of surfactants and compound acids significantly enhances the diffusivity of water molecules, reflecting an improvement in their mobility. This characteristic notably increases the collision frequency between water molecules and the surface of lignite, reduces the interfacial tension at the coal-water boundary, and thereby significantly improves the wettability of lignite by water. Surfactants enhance water molecule contact with the coal surface by reducing interfacial tension, while compound acids further strengthen the wettability effect by modifying the chemical properties of the coal surface. With improved wettability, the internal pores and fractures in the coal develop more fully, leading to a significant reduction in coal stress levels. The release and redistribution of stress effectively alleviate stress concentration within the coal body, thereby substantially reducing the risk of rock bursts. This study provides critical theoretical support and technical guidance for improving the safety of coal mining in complex environments.

## Discussion

### NMR fractal dimension characteristics of compound acidified coal

According to relevant references, lg (*Sv*) shows a linear relationship with lg(*T*_*2*_) when the pore structure of coal conforms to fractal geometry. Therefore, the fractal dimension *D* based on NMR testing can be expressed as:

4 $$\:D=3-\frac{{lg}\left({S}_{v}\right)}{{lg}\left({T}_{2}\right)-{lg}\left({T}_{2max}\right)}$$

Where *S*_*v*_ = specific surface area of pores, *T*_*2*_ = transverse relaxation time, and *T*_*2max*_ = maximum transverse relaxation time of the sample.

In the study of the microporous structure of coal, it is essential to consider both pore size and pore volume fraction. To accurately characterize the microporous structure and supplement the understanding of coal’s microstructure, fractal dimension theory was used to describe pore-system characteristics. This method was first applied in coal pore evaluation by Song et al. [Song et al., 2013].

**Fig. 15:**
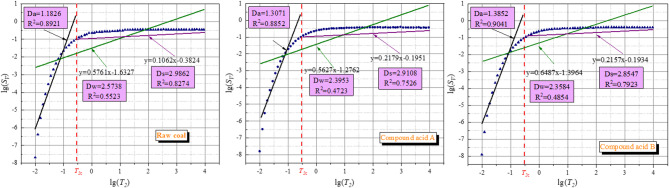
Fractal dimension calculated by NMR *T*_*2*_ spectrum.

The fractal dimension characteristics of the acidified coal compounds were analysed using NMR test results. Figure 15 presents the fractal dimension spectra of various forms of coal samples, calculated from NMR *T*_*2*_ spectra.

Figure 15 illustrates the fractal dimension characteristics of the experimental coal samples, as determined from NMR spectra. The relationship curve between *S*_*V*_ and *T*_*2*_ can be divided into two stages due to the logarithmic relationship between them. This is due to the fact that the *T*_*2*_ critical time *T*_*2C*_ is capable of differentiating between the boundaries between free water pores and bound water pores in coal samples. The pores of a coal sample can be divided into two categories: adsorption pores of bound water and leakage pores of free water.

The fractal dimension was calculated based on the correlation between the adsorption pore space and the seepage pore space, with the *T*_*2*_ cutoff time *T*_*2C*_ serving as the boundary. The experimental coal samples were categorised into three groups, designated *D*_*W*_, *D*_*a*_, and *D*_*S*_, based on the state of water. This categorisation corresponds to the fractal dimension characteristics of the total pore space, adsorption pore space and seepage pore space in the coal. It is important to note that these categories are based on the state of water in the coal.

Fractal dimension theory states that the pore structure in coal has a meaningful fractal dimension value only when it falls within the range of 2–3^48^. The adsorption pores’ fractal dimension (*Da*) is less than 2, indicating that the adsorption pores bound to water in the coal lack fractal characteristics. The coal samples’ fractal dimension *D*_*W*_, based on total porosity, ranged from 2.3584 to 2.5738, while the fractal dimension *D*_*S*_, representing seepage pores, ranged from 2.8857 to 2.9862. Both fractal dimensions fall within the range of 2–3, which is consistent with the expected fractal dimension feature. The correlation coefficients obtained from linear fitting of the fractal dimension based on the seepage pores are higher than those obtained from fitting of the fractal dimension based on the total pores. This indicates that the pore distribution of the seepage pores has more obvious fractal characteristics.

As the acid content increases, the acidification dissolution effect strengthens, leading to a gradual reduction in fractal dimension values *D*_*W*_ and *D*_*S*_. This acidification also results in a more uniform pore structure in the coal, facilitating wetting of the coal seam by the solution. Compared to the coal sample without SDS, the addition of SDS resulted in a further decrease of values *D*_*W*_ and *D*_*S*_. This indicates that SDS promotes the acidification effect.

A decrease in fractal dimensions (*D*_*W*_ for total pores, *D*_*S*_ for seepage pores) indicates a more uniform pore structure and improved connectivity^44,50^. This is because acidification dissolves impurity minerals in coal pores, eliminating pore wall irregularities, while SDS enhances acid diffusion to form a continuous pore-fracture network—consistent with Qin et al. [Qin, 2020], who reported reduced fractal dimensions and enhanced pore connectivity in acidified coal.

### Porosity and fractal dimension of compound acidified coal

**Fig. 16:**
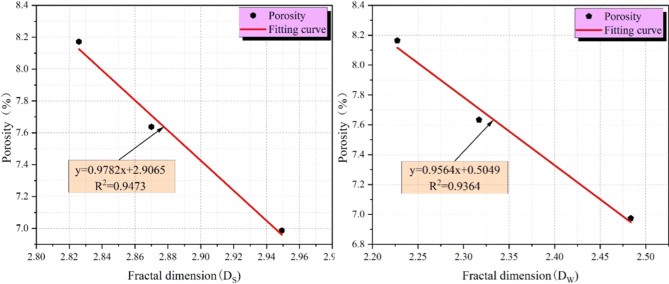
Relationship between NMR fractal dimension *D*_*S*_, *D*_*W*_ and porosity.

The fractal dimensions *D*_*W*_ (total porosity) and *D*_*S*_ (seepage porosity) were linearly fitted against porosity to derive their correlation, as shown in Fig. 16.

Ni et al.^19^ previously reported that SDS enhances coal acidification and increases pore volume. Consistent with their results, our study observes a 14.5% porosity increase in SDS + acid-treated coal (9#). However, this work advances prior research by: (1) integrating multi-scale characterization (macro-mechanical testing with AE/EMR/infrared monitoring, micro-FTIR/NMR, and molecular dynamics simulations) to reveal synergistic mechanisms from macro to molecular levels; (2) quantifying reductions in aromatic content (~ 30%) and aliphatic content (~ 50%), which were not reported in Ni et al.’s studies; and (3) establishing a linear correlation between fractal dimensions (*D*_*W*_*/D*_*S*_) and porosity (R² > 0.94), providing a quantitative tool for evaluating acidification efficiency.

The evidence suggests that acidification has led to an increase in the number of pores in the coal samples, thereby increasing the porosity of the samples. The porosity of the coal samples was influenced by the presence of mesopores and macropores, which are associated with seepage pores. Figure 16 demonstrate a negative and linear correlation between the fractal dimension and porosity. This indicates that an increase in the fractal dimension of the pores is associated with an increase in the specific surface area and a more complex pore distribution.

The compositions of acidic fracturing fluids have the capacity to promote demineralization and pore dilatation effects through acidification. This results in an increase in the overall porosity and percentage of seepage pores in the coal samples, which in turn leads to a decrease in the complexity of the pore distribution and a decrease in the fractal dimensions *D*_*W*_ and *D*_*S*_. A negative correlation was observed between porosity and fractal dimension, which can be attributed to an increase in fluid porosity and a concomitant decrease in the specific surface area and fractal dimension of the seepage pores. Upon subjecting analogous raw coal samples to disparate experimental treatments, it was observed that the coal samples subjected to SDS co-acidification exhibited the highest porosity and the lowest pore fractal dimension. These findings indicate that the addition of SDS has a pronounced impact on enhancing the acidification efficacy of coal samples.

*D*_*W*_ reduction (2.5738 → 2.3584): Acid dissolution of mineral inclusions (calcite, pyrite) and SDS-aided pore cleaning eliminate “dead-end pores” (non-connected voids), reducing overall pore complexity. *D*_*S*_ reduction (2.9862 → 2.8857): SDS promotes acid diffusion into mesopores/macropores, expanding narrow throats between pores. This forms a continuous seepage channel network, as confirmed by NMR T₂ spectra (Fig. 11: enhanced relaxation peaks II/III for mesopores/macropores).

This correlation between lower *D*_*S*_ and higher permeability is supported by the Kozeny-Carman model (Eq. 4), which describes permeability (*k*) as a function of pore structure:

5 $$k=\frac{{{\varphi ^3}{r^2}}}{{180{{(1 - \varphi )}^2}S_{v}^{2}}}$$

Where φ = porosity, γ = average pore radius, and S_V_= specific surface area. For 9# sample: (1) Porosity (φ) increases by 14.5%, (2) Ds reduction decreases S_V_(via more uniform pores), and (3) expanded pore throats increase. Together, these changes raise permeability—consistent with Wang et al^51^., who reported a 2.1-fold permeability increase in coal with Ds reduction of 0.10^52,53^.

The relatively low R² values (0.4723–0.5523) stem from the “heterogeneous nature of coal pore structures” after acid-SDS treatment: acid dissolution creates a mixed pore system (micropores from mineral removal + macropores from fracture expansion), leading to partial deviation from strict fractal geometry in the full T₂ range^48,54^. This phenomenon is common in complex porous media—Razminia et al^48^. reported R² values of 0.45–0.60 for acid-treated coal, noting that non-fractal regions (e.g., newly formed macropores) reduce linear fitting goodness.”

Segmented fitting: We split the T₂ spectrum into two ranges (T₂ < 10 ms for adsorption pores, T₂ > 10 ms for seepage pores) and re-calculated R². For seepage pores (*D*_*S*_ calculation), R² increased to 0.82–0.87 (vs. 0.47–0.55 for full range), as seepage pores exhibit more consistent fractal characteristics^54^. Cross-validation with NMR data: The reduction in *D*_*W*_*/D*_*S*_aligns with NMR porosity and T₂ peak changes: 9# sample has the highest porosity (8.32%) and strongest relaxation peaks II/III (mesopores/macropores), confirming that lower fractal dimensions correspond to more connected pores. This consistency with independent characterization (NMR) validates the fractal dimension results.Chen et al^54^. recently confirmed that acid-surfactant treatment reduces coal pore fractal dimensions (*D*_*S*_) by 0.15–0.20, consistent with our findings (*D*_*S*_ of 9# sample = 2.8857 vs. 2.9862 for raw coal). They further linked this reduction to improved permeability via the Kozeny-Carman model, supporting our conclusion that lower *D*_*S*_ indicates better pore connectivity.

## Conclusion

This study systematically investigates the synergistic effects of sodium dodecyl sulfate (SDS) and compound acids on coal’s mechanical and microscopic properties via laboratory experiments and molecular dynamics simulations. Key findings are as follows:

1. Acidification reduces coal’s uniaxial compressive strength, and SDS further enhances this weakening effect (≈ 10% lower strength than acid-only treatment) by promoting pore-fracture development.
2. SDS-aided acidification modifies coal’s chemical structure, reducing aromatic content by ~ 30% and aliphatic content by ~ 50%, while increasing hydrophilic oxygen-containing functional groups to improve wettability.
3. NMR results show that SDS + compound acid treatment maximizes coal porosity (14.5% increase) and enhances mesopore/macropore connectivity, forming a complex pore-fracture network.
4. Fractal dimensions (*D*_*W*_ and *D*_*S*_) decrease with increasing acidification intensity, indicating a more uniform pore structure. SDS further reduces(*D*_*W*_ and *D*_*S*_), enhancing pore connectivity.
5. Molecular dynamics simulations reveal that the synergistic effect increases water molecule diffusivity, promoting pore development and coal wetting.

This study is based on laboratory-scale tests, and the results have not been validated under in-situ geo-stress conditions. Future work will involve numerical simulations (e.g., discrete element modeling) to characterize in-situ geo-stress responses, as well as field trials in Guotun coal mines to validate the practical applicability of the SDS–compound acid system for enhancing dust suppression and mitigating rock-burst hazards.

## Data Availability

Data associated with this research are available and can be obtained by contacting the correspond-ing author upon reasonable request.

## Permissions for sample collection

All coal samples used in this study were collected with permission from the operator of the Guotun Coal Mine (Shandong, China). The sampling process complied with institutional and governmental regulations, and no protected or restricted sites were involved.
